# Deep Convolutional Neural Network for Flood Extent Mapping Using Unmanned Aerial Vehicles Data

**DOI:** 10.3390/s19071486

**Published:** 2019-03-27

**Authors:** Asmamaw Gebrehiwot, Leila Hashemi-Beni, Gary Thompson, Parisa Kordjamshidi, Thomas E. Langan

**Affiliations:** 1 Geomatics Program, Department of Built Environment, North Carolina A&T State University, Greensboro, NC 27411, USA; aagebrehiwot@aggies.ncat.edu; 2 North Carolina Emergency Management, Geodetic Survey, Raleigh, NC 27699-4298, USA; gary.thompson@ncdps.gov (G.T.); tom.langan@ncdps.gov (T.E.L.); 3 Computer Science Department, Tulane University, 6823 St. Charles Avenue, Tulane University, New Orleans, LA 70118, USA; pkordjam@tulane.edu; 4 Florida Institute for Human and Machine Cognition, Pensacola, FL 32502, USA

**Keywords:** remote sensing, convolutional neural networks, floodplain mapping, fully convolutional network, unmanned aerial vehicles, geospatial data processing

## Abstract

Flooding is one of the leading threats of natural disasters to human life and property, especially in densely populated urban areas. Rapid and precise extraction of the flooded areas is key to supporting emergency-response planning and providing damage assessment in both spatial and temporal measurements. Unmanned Aerial Vehicles (UAV) technology has recently been recognized as an efficient photogrammetry data acquisition platform to quickly deliver high-resolution imagery because of its cost-effectiveness, ability to fly at lower altitudes, and ability to enter a hazardous area. Different image classification methods including SVM (Support Vector Machine) have been used for flood extent mapping. In recent years, there has been a significant improvement in remote sensing image classification using Convolutional Neural Networks (CNNs). CNNs have demonstrated excellent performance on various tasks including image classification, feature extraction, and segmentation. CNNs can learn features automatically from large datasets through the organization of multi-layers of neurons and have the ability to implement nonlinear decision functions. This study investigates the potential of CNN approaches to extract flooded areas from UAV imagery. A VGG-based fully convolutional network (FCN-16s) was used in this research. The model was fine-tuned and a k-fold cross-validation was applied to estimate the performance of the model on the new UAV imagery dataset. This approach allowed FCN-16s to be trained on the datasets that contained only one hundred training samples, and resulted in a highly accurate classification. Confusion matrix was calculated to estimate the accuracy of the proposed method. The image segmentation results obtained from FCN-16s were compared from the results obtained from FCN-8s, FCN-32s and SVMs. Experimental results showed that the FCNs could extract flooded areas precisely from UAV images compared to the traditional classifiers such as SVMs. The classification accuracy achieved by FCN-16s, FCN-8s, FCN-32s, and SVM for the water class was 97.52%, 97.8%, 94.20% and 89%, respectively.

## 1. Introduction

Flooding is one of the leading threats of natural disasters to human life and property worldwide [1]. It is responsible for tens of thousands of deaths every year [2]. In the U.S., floods regularly cause catastrophic damage and human tragedy, especially in the urban environment. Flooding is often caused by extreme weather. It can be caused by heavy precipitation over a short period of time or continuous precipitation over several days. Flooding can also happen when ice or wind causes a river or stream to overflow their banks over the surrounding area. The 2016 U.S. National Weather Service (NWS) reported about 126 deaths and 10 billion dollars of property damage caused by flooding; the highest cause of weather-related deaths in the U.S. [3]. For example, in October 2016, Hurricane Matthew caused severe damage to the eastern parts of North Carolina, devastating towns such as Princeville, Lumberton, Smithfield, Kinston, Fayetteville, and Goldsboro. The damages associated with it were estimated at about $1.5 billion [4]. Hurricane Florence resulted in massive flooding in North and South Carolina, primarily as a result of freshwater flooding in September 2018. To mitigate the devastating effects of extreme weather events, many organizations (e.g., management agencies, policymakers, and local administration officials) need to have adequate early warning and preparation mechanisms and tools in place. Mapping of flooded areas during and immediately after flooding is critically important for flood emergency responses. Flood mapping can protect human lives and property damages by providing timely damage assessments to plan relief work efficiently.

The use of remote sensing data to produce inundation mapping and to assess flood hazards in near real-time has been popular over the last few decades [5]. Flood maps can be prepared using data from satellites, aircraft, and Unmanned Aerial Vehicles (UAVs). Several researchers studied flood risk assessment using satellite and aerial images for large-scale projects [6,7,8]. UAV technology can sufficiently generate faster and more accurate data at much lower costs for rapid flood assessment. UAVs can effectively acquire high-resolution data for fast and accurate detection of inundated areas under complex urban landscapes as well as inaccessible areas due to hazardous environments as compared to other data acquisition approaches [9,10]. UAVs also have a low dependency on launching and landing conditions, which makes them safer and more applicable compared to piloted aircrafts in urban flood monitoring.

Accurate and immediate extraction of the flood extent is very important for immediate emergency response. Various image classification and segmentation techniques such as Support Vector Machines (SVMs) have been used to extract flooded areas [11]. SVMs perform well for remote sensing applications where a limited amount of data is used, however, the complexity grows as the number of training samples increases. In recent years, different deep learning approaches including Deep Belief Networks (DBN) [12], Convolutional Neural Networks (CNN) [13], and Recurrent Neural Networks (RNN) [14] have been applied for image classification, object detection, and recognition tasks. CNNs are widely used in image classification because of their ability to successfully handle large training data sets, often achieving higher classification accuracy than the traditional methods [15,16]. CNNs can learn features automatically from the large datasets through the organization of multi-layers of neurons [17] and have the ability to implement nonlinear decision functions [18].

Many researchers have studied CNN-based image segmentation methods such as Fully Convolutional Network (FCN) [19], SegNet [20] and U-Net [21]. Among these models, FCNs have been used for many studies in Remote Sensing. FCN was proposed by Long et al. [19] to train an end-to-end for semantic segmentation. In this model, VGG16 fully connected neural layers were replaced by convolutional neural layers to maintain the 2-D structure of images. The FCN model took images of arbitrary size as inputs and generated correspondingly sized output with efficient inference and learning and achieved classification accuracy higher than 80%. Marmanis et al. [22] and Sherrah et al. [23] employed the FCN model to perform a semantic segmentation for traditional aerial imagery while Fu et al. [15] used the model to classify high-resolution satellite images with the average precision, recall, and Kappa coefficient of 0.81, 0.78, and 0.83, respectively. Nguyen et al. [24] presented a five-layered network algorithm for satellite image classification, and achieved an average classification accuracy of 83% using six classes. Castelluccio et al. [25] adopted Cafenet and Googlenet CNN architecture with three different learning modalities for land use classification of remote sensing images and achieved an overall accuracy of about 91%. There have also been some studies on the application of deep convolutional neural network to classify hyperspectral images that provide a better classification performance compared to SVMs [16,26]. 

Despite the research studies on CNN method for the classification of remote sensing data, CNN techniques for segmentation of flooded regions from UAV data is rare and has not been well documented. Based on that context, in this paper, we investigated the FCN16s method based on transfer learning for inundation area extraction. By transfer learning here we meant the reusing of a pre-trained model for a new problem. This technique provided an effective way of training a large network using our UAV training data without having an overfitting issue. We fine-tuned a pre-trained FCN-16s model to extract flooded areas. This approach was tested on the UAV imagery captured over three flood-prone study areas in North Carolina, United States immediately after Hurricane Matthew and Hurricane Florence, respectively. To assess the performance of the model, the results of the FCN-16s classifier was compared to SVM, FCN-8s and FCN-32s classifiers. 

This paper is organized as follows. The data used for the research and study area is described in Section 2. Section 3 provides an overview of the most relevant components of the convolutional neural network and our proposed network architecture. The method to train classifiers based on CNNs and the experiments is described in Section 4. The experimental results and discussions are presented in Section 5. The conclusion and outlook for future work is highlighted in Section 5.

## 2. Methods

The approach used in the research was based on the concept of transfer learning where a CNNs model is trained based on one dataset and can be transferred and used to classify another dataset to efficiently solve the image segmentation problem. We fine-tuned FCN-16s for UAV-based image segmentation for flood extent mapping. Our approach consisted of labeling, training, classification and accuracy assessment stages. Labeling is an important stage in any supervised image segmentation task that needs training data. In the labeling stage, each pixel in an image is assigned to a predefined class. In the training stage, the UAV images and its corresponding pixel-labeled images are uploaded onto the CNNs network as a training dataset. The approach evaluated the model based on a 10-fold cross-validation. In the classification stage, the trained network was applied to an input image to predict multiple classes. The network learned to associate image segments and labels during training, and predicted the class labels for the test set. In the accuracy assessment stages, a confusion matrix was generated to measure the classification accuracy. The following sections describe CNNs and the proposed method in detail. 

### 2.1. Convolutional Neural Networks

CNNs are a type of feed-forward artificial neural network made up of layers that have learnable parameters including weights and biases. The concept of CNNs was first proposed by Fukushima et al. [27] and refined by LeCun et al. [28]. The network was trained by a back propagation algorithm [12]. Backpropagation is a supervised learning technique used in an Artificial Neural Network (ANN). It optimizes the parameters of the neural network (i.e., weights) based on the gradient descent technique. CNNs are widely used in machine learning to solve large scale problems concerning computer vision, natural language processing, pattern and speech recognition. CNNs are trainable multi-layer network structures, which are composed of multiple feature-extraction stages. Each feature-extraction stage of CNNs is composed of convolutional layers, pooling layers and nonlinearity layers or activation functions: 

(A) Convolutional layers: A convolution layer is the first layer to extract features, such as edges or textures from an input image. It applies a convolution operation to the input, passing the result to the next layer. The input can be a three-dimensional (3D) m × n × r image where m is the height, n is the width of the image, and r is the number of channels. The output of the convolution layer is a feature map. The convolutional layer computes the output feature map by summing up all of the neuron’s input values $\left(x_{i}\right)$, the weighted inputs (w_*ij*_) of the neuron plus the bias parameter (b_*j*_) and then applying an activation function on it:

(1) $$y=\sum_{i=1}^{n}w_{ij}*x_{i}+b_{j}$$

where * is a two-dimensional discrete convolution operator.

(B) Pooling layers: A pooling layer is a down-sampling layer, which commonly comes after each convolution layer and takes feature maps as its input. The main purpose of this operation is to progressively reduce the spatial size of the feature maps to reduce the number of parameters and computation in the network, and thus to control overfitting. 

(C) Activation function: This function basically decides whether or not a neuron should be activated, in other words, whether the information that the neuron is receiving is relevant for the given information or whether it should be ignored. Activation functions introduce non-linearity to a network. Different activation functions have been used for different problem setting contexts including: Sigmoid function, Hyperbolic tangent function, and Rectified Linear Unit (ReLU) function. A sigmoid function takes a real-valued number and maps it into a range between 0 and 1. In particular, large negative numbers become 0 and large positive numbers become 1. The hyperbolic tangent function is similar to the sigmoid function but the output values range between −1 and 1. ReLU or Rectified Linear Unit function is linear (identity) for all positive values, and zero for all negative values; defined as:

(2) f(x)=max(0,x)

A fully connected layer is used to connect all values in the input to the final classification results and finally, Softmax layers are used to calculate the final probabilities of each class. 

### 2.2. Fully Convolutional Network

**Figure 1:**
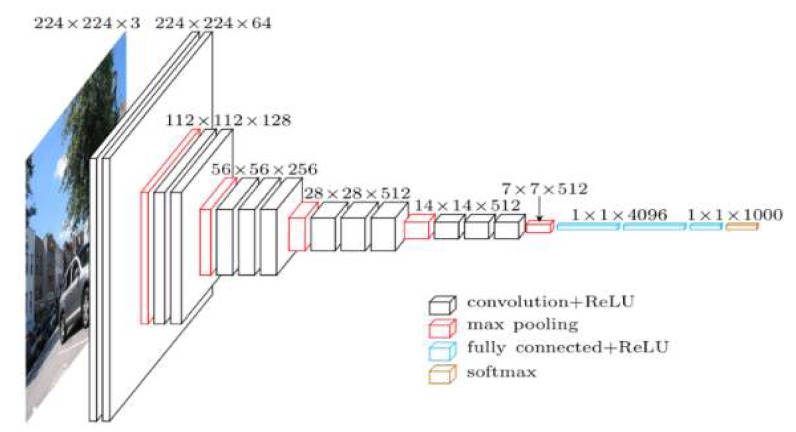
**VGG-16** Architecture [29,31].

**Figure 2:**
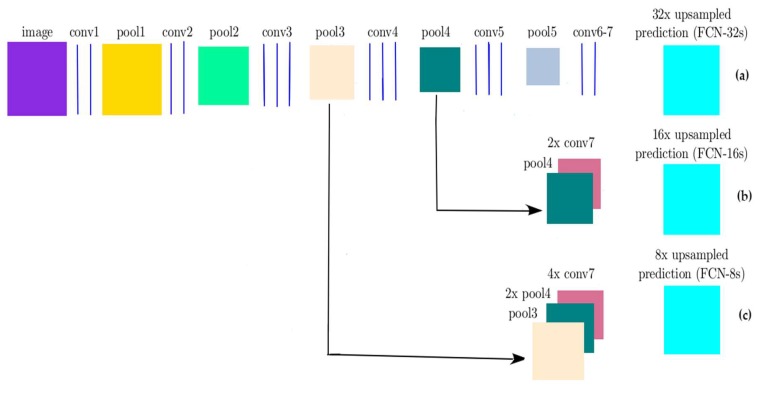
FCN architecture: (**a**) FCN with an output stride 32 (FCN-32s); (**b**) FCN with an output stride of 16 (FCN-16s); (**c**) FCN with an output stride of 8 (FCN-8s) [19].

Different network architectures have been proposed and developed for image classification [29,30]. Simonyan et al. [29] designed a deep CNN network called VGG. This architecture was developed to increase the depth to 16 to 19 weight layers while making all filters with at most 3 × 3 sizes to reduce the number of parameters in the network. VGG16 is composed of 13 convolutional layers with 3 × 3 receptive fields with stride and padding of 1, five max pooling layers of size 2 × 2 pixel window with a stride of 2, followed by three fully connected layers, and the soft-max layer (Figure 1). In this network, the fully connected layers require a fixed image dimension to connect the values in the input to the final classification results. The first two fully connected layers have 4096 channels each, and the third layer contains 1000 channels. The classification output of a fully connected-based CNN network like VGG16 is a one-dimensional (1D) feature vector. However, for our study, a 2-dimensional (2D) class map is always needed. This is accomplished by replacing fully connected layers of VGG (two layers with 4096 neurons and one with 1000 neurons) with convolutional layers and adding deconvolutional (upsampling) layers, leading to an FCN network. Therefore, in this study, the FCN network with an output stride of 16 (FCN-16s) [19] was used, in which fully convolutional layers were made up by transforming the fully-VGG connected layers to convolutional layers, while preserving the learned parameters. FCN was constructed only from locally connected layers, such as convolution, pooling and upsampling. The FCN architecture did not include a fully connected layer. FCN-16s combined the 2× upsampled prediction starting from conv7 with pool4 to generate a segmentation result at stride 16 (Figure 2). Each of these pooling layers downscaled the input by a factor of two horizontally and vertically. The last convolutional layer and two intermediate layers were followed by deconvolutional layers that up-sampled the network output to the size of the input image. In this research, we investigated the performance of a FCN model to classify high-resolution UAV imagery for flood application. In the training phase, the number of output classes of FCN-16s was modified from 21 classes to 4 classes (water, building, vegetation, and road) to use for training our dataset. 

**Figure 3:**
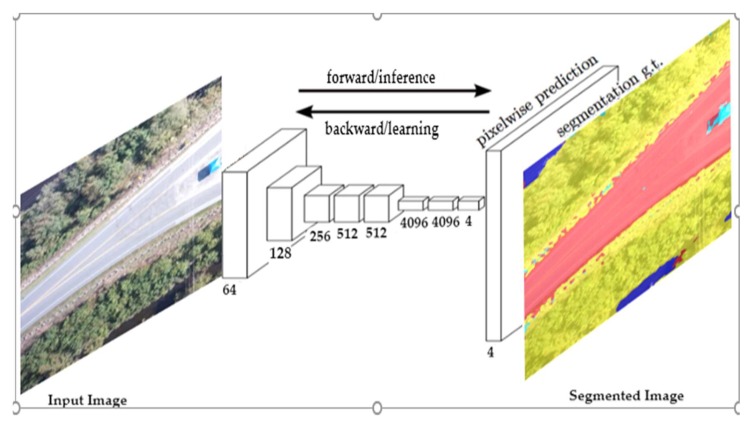
FCN-16s architecture with four classes [19].

The VGG16 model was trained on more than a million images from the ImageNet database. Training the network from scratch with limited training data usually generates poor results, and often results in overfitting. In this study, a small dataset was available (100 images). Moreover, the images were captured from UAV, which was different from the pre-trained model’s dataset, hence, we needed to train an FCN-16s network with pre-trained weights assigning a reduced learning rate (fine-tuning). Fine-tuning is the process of training CNNs in a different dataset, which is usually much faster and easier than training CNNs with randomly initialized weights from scratch. A schematic of FCN-16s based network architecture is shown in Figure 3. 

## 3. Experiments

### 3.1. Study Area and Data

**Figure 4:**
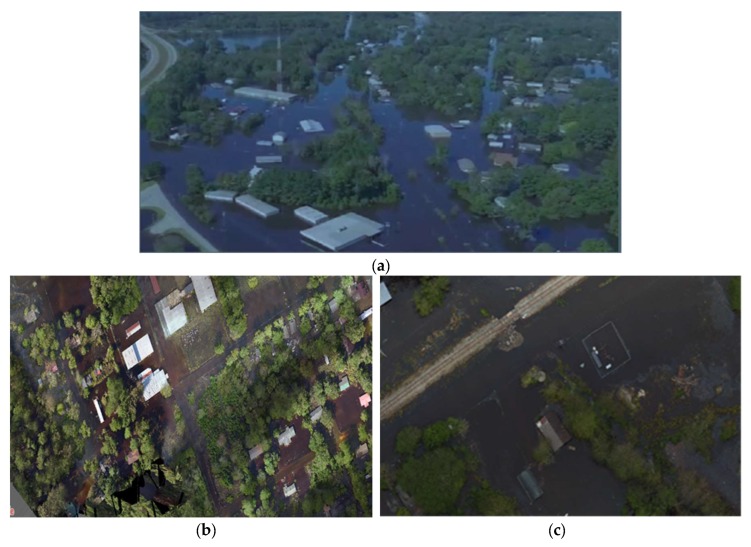
The study areas: (**a**) Princeville (**b**) Lumberton and (**c**) Fair Bluff City.

Three flood-prone areas in North Carolina, USA were selected for the research. The study areas are shown in Figure 4:

(1) The town of Princeville in Edgecombe County during a flooding event as a result of Hurricane Matthew in October 2016.
(2) The city of Lumberton in Robeson County during a flooding event as a result of Hurricane Florence in September 2018.
(3) The city of Fair Bluff in Columbus county during a flooding event as a result of Hurricane Florence in September 2018.

In October 2016, North Carolina Emergency Management (NCEM) collected aerial imagery of areas flooded by hurricane Matthew over Princeville using a Trimble UX5 fixed-wing UAV. Each image consisted of three bands (RGB) with 2.6 cm spatial resolution and 10,816 m^2^ land coverage. The UAV data in Lumberton and Fair Bluff was collected by using a DJI M600 UAV immediately after Hurricane Florence in 2018. Each image consisted of three bands (RGB) with 1.5 cm spatial resolution and 1159 m^2^ land coverage.

### 3.2. Labeling Stage

In this stage, we manually labeled a total of 100 RGB UAV images of size 4000 × 4000 pixels; 70 images from the city of Princeville and 30 images from the city of Lumberton and Fair Bluff. The Matlab image labeler application was used to classify each pixel into water (blue), building (red), vegetation (yellow), and road (purple). First, the Flood Fill, which is a semi-automated tool, was used to label a group of connected pixels that had similar color such as water, building, vegetation or building in our case and then the labels were manually refined. Thus, for each image, there was a 4000 × 4000 label map having a pixel-class correspondence with it. After labeling our data, the labels were exported as ground truth data for training. It required approximately 13 h to label 100 UAV imagery (water, building, road and vegetation).

It should be noted that not all training images contained four kinds of pixel classes (i.e., water, building, vegetation and road). We applied the median frequency balancing method to deal with the imbalance problem. In this method, the weight assigned to each class in the loss function was the ratio of the median of the class frequencies computed on the entire training set divided by the class frequency. The class frequency was calculated by dividing the number of pixels for each class by the total number of pixels in the image. In the training data, the frequency for the classes of water, building, vegetation and road was calculated as 41.1%, 5.6%, 9% and 44.3% respectively. 

### 3.3. Training and Classification Stage

UAVs imagery has a large size and contains rich information about the area. During the training stage, the full-size images are not inserted to the FCN-16s due to the memory limitation. Therefore, the original images are split into a patch size of 500 × 500. A learning rate of 0.0001, and maximum epoch of 6 are used for all classes. To estimate the performance of the classification, we trained the network with a k-fold cross validation procedure. The purpose of this evaluation procedure was to avoid overfitting the data and improve the generalization performance of the CNNs model. For this, we partitioned the data (100 images) randomly into 10 equal subsets, called folds. At each run, the union of nine folds were put together to form a training set, and the remaining one fold used as a testing or validation set to measure the classification errors. We repeated the above steps 10 times, using a different fold as the testing set each time. In other words, the data included in the first validation fold would never be part of a validation fold again. Finally, the average error from all 10 folds was used to estimate the classification errors. During this stage, the final layer was trained from scratch, while the others were initialized from the pre-trained model and updated by the back-propagation rule. The training stage ended after 176,500 iterations for all 10-fold experiments. In the classification stage, the trained network was applied on an input image to generate a class prediction. The network learned to associate images and labels and make a prediction about the test dataset to generate the final classification output. It required approximately 26 h for cross validation using a single GPU (NVIDIA Quadro M4000).

### 3.4. Accuracy Assessment Stage

In this research, we used a Confusion matrix [32] to analyze the accuracy of the classification method. A confusion matrix evaluates the performance of a classification model on a set of test data for which the true values are known. A confusion matrix provides detailed information on how each classifier is performing. In addition, kappa coefficient was used in this study to summarize the information provided by the confusion matrix.

To further verify the performance of the FCN-16s method, the classification results were compared with the results of other classification methods namely FCN-8s, FCN-32s and SVM.

#### 3.4.1. FCN-8s and FCN-32s

We trained the FCN-8s and FCN-32s models [19] using the same set of training images used for the FCN-16s method. Figure 3 shows the difference between FCN-8s, FCN-32s, and FCN-16s architectures. We applied the FCN-8s, and FCN-32s to classify high-resolution UAV imagery with four classes (water, building, vegetation, and road) and fine-tuned the network based on the ImageNet pre-trained model. The same training parameters of FCN-16s were applied for the FCN-8s and FCN-32s and 10-fold validation was used to evaluate the performance of the model. 

#### 3.4.2. Support Vector Machine

To further evaluate the performance of our FCNs over the traditional learning approaches, a pixel-based SVM classification [33] was considered to extract flooded areas from UAV images. SVM is a supervised classifier proposed by Vapnik [34], which is based on statistical learning theory and is commonly used for aerial image classification [35,36]. The SVM classifier works by mapping the training sample data into a highly dimensional feature space, and finds the best hyperplane that separates all data points of one class from another class. It separates samples belonging to different classes by tracing maximum margin hyperplanes in the kernel space where samples are mapped. A margin is the maximal width of the slab parallel to the hyperplane that has no interior data points. In this study, about 20% of randomly selected pixels in each class were used as a training (labeled) sample. 

## 4. Results

### 4.1. FCN-16s

**Figure 5:**
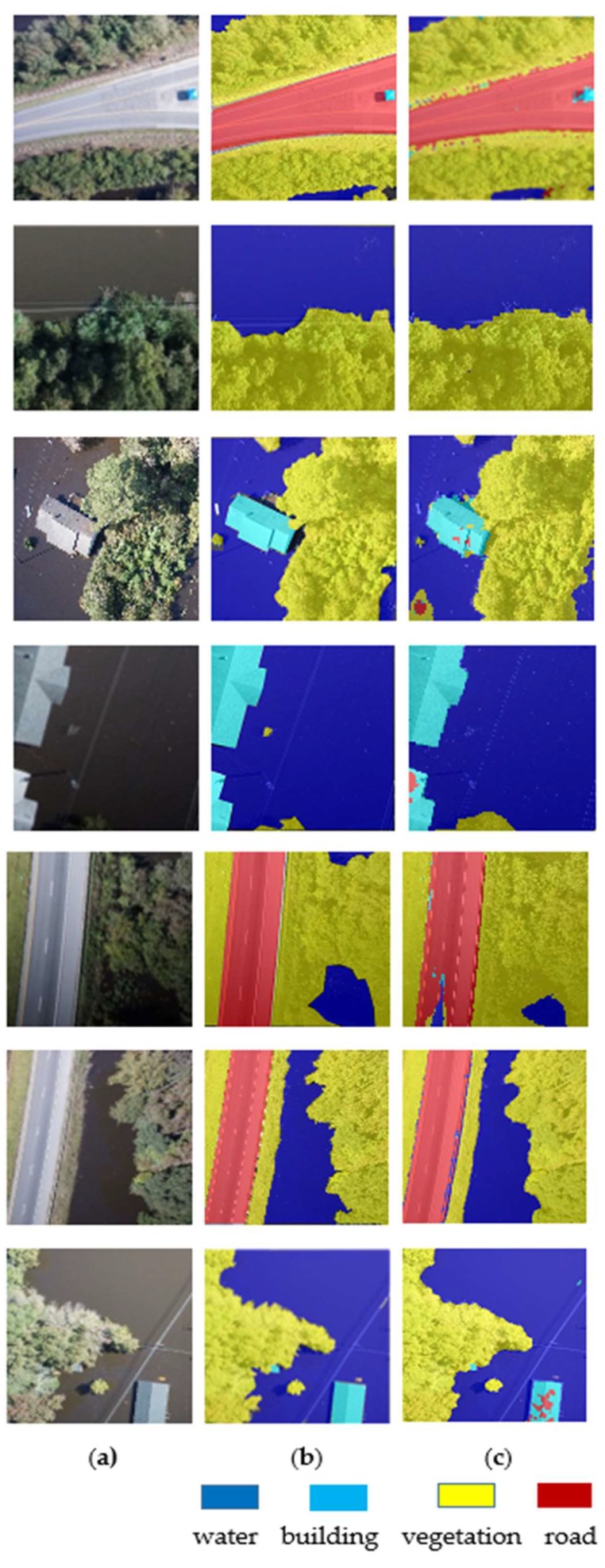
Classification results of the FCN-16s. (**a**) Original RGB images; (**b**) labelled images (ground truth); (**c**) results of FCN-16s classification.

The FCN-16s, FCN-8s, FCN-32s, and SVMs were implemented in MATLAB. The sections below describe the results of classifying the UAV imagery in support of flood management. The classification results of FCN-16s are shown in Figure 5 and the detailed information on how each classifier (for water, building, road and vegetation classes) performed is described in Table 1, via the confusion matrix. The overall accuracy and kappa index achieved for the FCN-16s method was about 95% and 0.904 respectively. 

The cells of the confusion matrix show the percentage of correct and incorrect prediction for all the possible correlations between the known reference data (ground truth) and the classified image. The cell in the ith row and *j*th column means the percentage of the *i*th class samples which were classified to the *j*th class. For example, 97.52% of class water samples were classified correctly, but 1.398% of class water samples were incorrectly classified as the building class. The diagonal cell of the matrix contained the number of correctly identified pixels for each class. Our goal in this study was to extract flooded areas (water class) from the UAV imagery. The FCN-16s method achieved about 97.5% accuracy in extracting the flooded area or water class. 

### 4.2. Comparison between Classifiers

The FCN-8s, FCN-32s, and SVM classifiers were also implemented in MATLAB to classify the UAV images for flooded extent mapping. Table 2 compares the results of classifiers for four classes: Water, building, vegetation and road.

**Figure 6:**
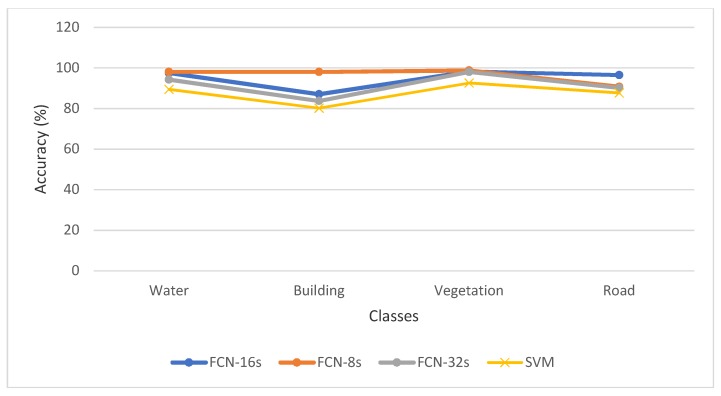
Accuracy analysis for each of the individual classes.

Figure 6 shows the accuracy of the classification methods for each of the classes. The results showed that the FCNs had a better performance than the traditional SVM classifier in the UAV images segmentation. Among the FCNs, FCN-8s performed better than FCN-16s and FCN-32 as it captured more hierarchical features [19]. 

For the flood management and damage assessment applications, UAVs generally collect a large dataset of high temporal and spatial resolution images, thus, handling of the large training data sets is critical for an accurate flood extent mapping. FCNs are more suitable for the segmentation of large data sets [15] compared to SVMs where the training complexity of SVMs is highly dependent on the size of the data set. FCNs learn features automatically from the large dataset through the organization of multi-layers of neurons [17] and have the ability to implement nonlinear decision functions [18]. 

## 5. Conclusions and Future Works

UAVs technology is an efficient photogrammetry data acquisition platform that can be used to quickly deliver high-resolution imagery for flood assessment and emergency response. In this paper, we investigated a deep learning approach to extract the flooded areas from high-resolution UAV imagery. Training a deep CNN from scratch with a small dataset is not always advisable due to poor classification results, and overfitting. Adapting pre-trained models and properly fine-tuning them provided promising results for UAV imagery classification. In this study, the FCN-16s model was fine-tuned and trained to extract the inundated areas. FCN-8s model was trained using the same dataset and parameters used for the FCN-16s network. To test the performance of convolutional neural networks over traditional classifiers, the pixel-based SVM classification approach was implemented. Experimental results indicated that a CNN-based classifier such as FCN-16s was very suitable in flood imagery segmentation with an overall accuracy of 95% and a Kappa index of 0.904. Overall, the experimental results showed that we managed to achieve promising classification results even though only one hundred UAV images were available for training. There are still some challenges for using the deep learning method for mapping the water extent under a canopy as well as in shadows where the terrain (or flooded area) is masked by high vegetation or shadows [37]. These issues would be reduced by performing a 3D terrain analysis and combining the results with those of image segmentation assuming that the surface of the water (lakes and in our case, flooded areas) are flat. 

## Figures and Tables

**Table 1:** Confusion matrix for the FCN-16s (unit: Percentage).

	Water	Building	Vegetation	Road
Water	97.520	1.398	1.032	0.053
Building	7.789	87.043	2.723	2.445
Vegetation	1.220	0.444	98.249	0.087
Road	0.346	2.199	0.954	96.500

**Table 2:** Overall accuracy and Kappa index for FCN-16, FCN-8, FCN-32s and Support Vector Machine classifiers.

Title 1	Overall Accuracy	Kappa Index
FCN-16s	95.000 %	0.904
FCN-8s	95.520%	0.912
FCN-32s	92.000%	0.870
SVM	87.450%	0.790
